# Kinetic and Thermodynamic Study of Proton Transfer Reactions of 1‐Hydroxy‐2,2‐Dinitroethane: Establishing a Predictive Relationship between Tautomeric and Acidity Constants in Water

**DOI:** 10.1002/cphc.202500219

**Published:** 2025-07-23

**Authors:** Rania Khaldi, Amel Hedhli, Taoufik Boubaker

**Affiliations:** ^1^ Laboratoire de Chimie Hétérocyclique Produits Naturels et Réactivité (LR11ES39) Faculté des Sciences de Monastir Université de Monastir Avenue de l’Environnement 5019 Monastir Tunisie

**Keywords:** 1‐hydroxy‐2,2‐dinitroethane, brönsted–Marcus approach, intrinsic reactivity, kinetics, nitroalkanes, proton transfer

## Abstract

The proton transfer reactions of the nitronate anion derived from 1‐hydroxy‐2,2‐dinitroethane **1** with HCl and carboxylic acid buffers in aqueous solution at 25 °C are investigated through a comprehensive kinetic and thermodynamic analysis. The reaction mechanism is elucidated, allowing the determination of acidity constants for both O‐protonation ($pK_{a}^{{\text{NO}}_{2}H}$ = 1.67) and C‐protonation ($pK_{a}^{\text{CH}}$ = 3.78), which are among the weakest reported for nitroalkanes in water to date. The intrinsic rate constant (log *k*_o_ = 1.60), determined using the Marcus approach, is markedly lower than typical values reported for nitrile protonation, reflecting the exceptional resonance stabilization of the conjugate base by the strongly electron‐withdrawing NO_2_ groups. Most importantly, by combining reported data from the literature with the results obtained in this study, a fundamental and predictive relationship between the tautomeric equilibrium constants (p*K*_N_ = $pK_{a}^{\text{CH}}$‐$pK_{a}^{{\text{NO}}_{2}H}$) and the acidity constants ($pK_{a}^{\text{CH}}$) of nitroalkanes in water has been established. This unprecedented linear correlation allows to estimate the acidity constants of the nitronic acids of four nitroalkanes in water, which were previously experimentally inaccessible. To the best of knowledge, this work is among the first to exploit such an approach, representing a significant advancement in understanding the structure–reactivity relationships governing the protonation of carbon and oxygen sites in nitroalkanes.

## Graphical abstract

Kinetic and thermodynamic analysis of 1‐hydroxy‐2,2‐dinitroethane nitronate reveals very low acidity constants for O‐ ($pK_{a}^{{\text{NO}}_{2}H}$ = 1.67) and C‐protonation ($pK_{a}^{\text{CH}}$ = 3.78) in water. Marcus theory modeling indicates that NO_2_ resonance stabilization significantly retards protonation. Original linear correlation between tautomeric (p*K*_N_) and acidity constants ($pK_{a}^{\text{CH}}$) in nitroalkanes is established, enabling prediction of previously inaccessible nitronic acid $pK_{a}^{{\text{NO}}_{2}H}$ values and providing deeper insight into protonation behavior.

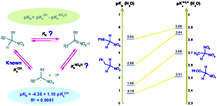

## 1 Introduction

**Scheme 1:**
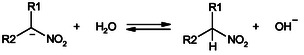
C‐protonation equilibrium of nitroalkane (R_1_R_2_CHNO_2_) and its conjugate nitronate anion (R_1_R_2_CNO_2_^−^) in water.

Protonation plays a fundamental role in determining the reactivity and stability of organic anions, influencing key transformations in both synthetic and biological systems. Among these, the protonation dynamics of nitronate anions are particularly relevant in organic and bioorganic chemistry.^[^ ^1^, ^2^ ^]^ A key question is whether protonation occurs at the carbon or oxygen site, as this selectivity directly impacts reaction pathways and intermediate stability. Under neutral conditions, protonation of nitronate anions (R_1__2_CNO_2_^−^) by water is typically assumed to proceed via C‐protonation, following the principle of microscopic reversibility (**Scheme** **1**).^[^ ^3^, ^4^, ^5^, ^6^, ^7^, ^8^, ^9^ ^]^

**Scheme 2:**
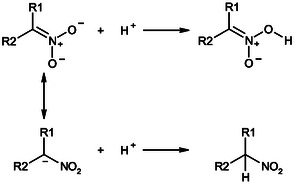
Protonation of the nitronate anion under acidic conditions, illustrating the competition between C‐ and O‐protonation.

However, experimental studies in acidic media indicate a shift in site selectivity, with protonation preferentially occurring at the oxygen site. This highlights the crucial role of proton donor acidity in governing site selectivity. Both C‐protonation and O‐protonation have been observed in various solvents, with measured acidity constants revealing the differential stability of the protonated forms.^[^ ^10^, ^11^, ^12^, ^13^, ^14^ ^]^ These findings emphasize the complexity of protonation mechanisms, which are dictated by the acidity of the medium as well as the intrinsic electronic properties of the intermediates involved. A deeper understanding of this competition is essential for rationalizing reaction mechanisms and designing selective protonation strategies (**Scheme** **2**).

Proton transfer reactions of nitroalkanes are well known to exhibit atypical kinetic behavior, a phenomenon commonly referred to as the “nitroalkane anomaly”. This refers to unexpectedly fast proton transfer rates compared to those predicted based on acidity or basicity alone. The origin of this anomaly has been extensively studied by Yamataka and coworkers,^[^ ^13^ ^]^ who demonstrated that early transition states and significant charge delocalization in the conjugate base play key roles in this behavior. These findings provide valuable insight into the reactivity patterns of nitroalkanes and highlight the importance of electronic and structural factors beyond simple acidity constants.

Despite extensive studies on nitronate chemistry, no systematic investigation has yet been dedicated to establishing a potential correlation between the acidity of nitronic acids (p*K* 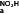) and that of their corresponding nitroalkanes ($pK_{a}^{\text{CH}}$). Bernasconi and collaborators attempted to correlate intrinsic reactivity (log *k*_o_) with the acidity constants ($pK_{a}^{\text{CH}}$) of various nitroalkanes.^[^ ^11^ ^]^ The weak correlations observed were attributed to a complex interplay of electronic and steric effects, including resonance stabilization, inductive and field effects, steric hindrance, polarization, solvation, and hyperconjugation. These results underscore the intricate relationship between the kinetic term, defined by intrinsic reactivity (log *k*_o_), and the thermodynamic term, measured by the acidity constant ($pK_{a}^{\text{CH}}$). They also highlight the necessity for a more in‐depth exploration of these interdependencies. In this context, a fundamental question arises: Is there an intrinsic relationship between the two acidity scales of nitroalkanes, namely $pK_{a}^{\text{CH}}$ and $pK_{a}^{{\text{NO}}_{2}H}$?

**Scheme 3:**
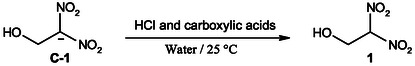
Reactions of **C‐1** in HCl and various carboxylic acid buffers in aqueous solutions.

To address this question, we investigated the kinetics of protonation of the sodium salt of 1‐hydroxy‐2,2‐dinitroethanide **C‐1** by hydrochloric acid and various carboxylic acid buffer solutions in aqueous media (**Scheme** **3**). These studies revealed the different protonation pathways and the corresponding acidities of the protonated forms. Most importantly, by combining our experimental results with literature data, we established a fundamental and predictive relationship between the tautomeric equilibrium constants (p*K*_N_) and the acidity constants ($pK_{a}^{\text{CH}}$) of nitroalkanes in water. This unprecedented linear correlation enabled us to estimate the acidity constants of four nitronic acids that were previously experimentally inaccessible. To the best of our knowledge, this work represents one of the first attempts to exploit such an approach, marking a significant advancement in understanding structure–reactivity relationships governing the protonation of carbon and oxygen sites in nitroalkanes. The results not only provide new insights into protonation equilibria but also offer a predictive tool for related systems, paving the way for broader applications in organic reactivity and catalysis.

## 2 Results

### 2.1 Protonation Reactions of 1‐Hydroxy‐2,2‐Dinitroethanide C‐1 under Hydrochloric Acid Solutions

**Figure 1:**
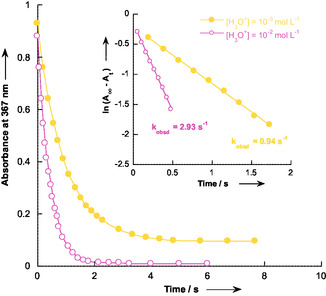
Plots of the absorbance at *λ*_max_ = 367 nm versus time for the protonation of **C‐1** (3 × 10^−5^ mol L^−1^) with H_3_O^+^ (10^−2^ and 10^−3^ mol L^−1^) and correlations ln (*A*_∞_ − *A*_t_) versus time at 25 °C in aqueous solution and *I* = 0.1 mol L^−1^ KCl.

The protonation kinetics of 1‐hydroxy‐2,2‐dinitroethanide **C‐1** were studied in water at 25 °C, maintaining a constant ionic strength of 0.1 mol L^−1^ with KCl. The reaction was monitored using a stopped‐flow spectrophotometer by measuring the decrease in absorbance at the absorption maximum of C‐1 (*λ*_max_ = 367 nm, see Figure S1, Supporting Information). The experiments were conducted under pseudo‐first‐order conditions, with a large excess of hydrochloric acid relative to the concentration of carbanion **C‐1** ([C‐1] ≈3 × 10^−5^ mol L^−1^). Throughout the experiments, only one relaxation process, corresponding to the formation of acid **1**, was observed, allowing the determination of the first‐order rate constants *k*_obsd_ (See **Figure** **1** for a representative example). The observed pseudo‐first‐order rate constants, *k*_obsd_, obtained under different experimental conditions, are summarized in **Table** **1**.

**Table 1:** Observed pseudo‐first‐order rate constants, *k*_obsd_, for the protonation of carbanion **C‐1** in HCl solutions in water at 25 °C and constant ionic strength of 0.1 mol L^−1^ maintained with KCl.

[H^+^] [mol L^−1^]	*k*_obsd_ [s^−1^]	[H^+^] [mol L^−1^]	*k*_obsd_ [s^−1^]
1 × 10^−3^	0.94	3.5 × 10^−2^	6.20
4 × 10^−3^	1.80	4 × 10^−2^	6.45
6 × 10^−3^	2.13	5 × 10^−2^	6.72
1 × 10^−2^	2.93	6 × 10^−2^	6.83
1.5 × 10^−2^	3.80	7.5 × 10^−2^	6.89
2 × 10^−2^	4.58	8 × 10^−2^	6.91
3 × 10^−2^	5.77	10^−1^	6.90

### 2.2 Proton‐Transfer Reactions of 1‐Hydroxy‐2,2‐Dinitroethanide C‐1 in Carboxylic Acid Buffer Solutions

**Scheme 4:**
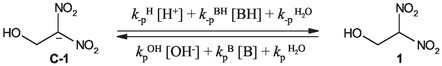
Proton‐transfer equilibrium reactions of 1‐hydroxy‐2,2‐dinitroethanide **C‐1** in carboxylic acid buffer solutions in water at 25 °C and *I* = 0.1 mol L^−1^ KCl.

The rates of the reversible protonation of the carbanion **C‐1** according to the general **Scheme** **4** were measured at a controlled temperature of 25 °C in aqueous solution, using carboxylic acid buffer solutions to maintain a stable pH environment. Throughout the experiments, the ionic strength was carefully controlled at *I* = 0.1 mol L^−1^ by adding KCl. The rates were measured photometrically in water at 25 °C by a stopped‐flow method under pseudo‐first‐order conditions, maintaining a large excess of the buffer, acid, or base reagent relative to the concentration of **C‐1** or **1** (3 × 10^−5^ mol L^−1^)

Under these experimental conditions, the observed rate constants, *k*_obsd_, were determined to describe the approach to equilibrium in the protonation process. These rate constants can be expressed through the following general equation (Equation (1)), which takes into account various factors influencing the rate of protonation and deprotonation. In this equation, the constants 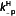, 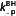, and 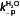 represent the contributions from the protonation of the carbanion **C‐1** by hydronium ions (H_3_O^+^), the acidic form of the buffer, and the solvent molecules. Meanwhile, the constants $k_{p}^{\text{OH}}$, $k_{p}^{B}$, and $k_{p}^{H_{2}O}$ correspond to the deprotonation of acid **1** by hydroxide ions, the base species of the buffer, and the solvent, respectively. (Table S1–S7, Supporting Information) summarize the observed pseudo‐first‐order rate constants, *k*_obsd_, measured under various experimental conditions. These rate constants are expressed by the general equation (Equation (1)), which accounts for the contributions of different reactive species.
(1)

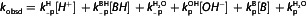




The analysis of the data in (Table S1–S7, Supporting Information) in accordance with Equation (2), confirmed that the plots of *k*_obsd_ versus [*BH*] or [*B*] at constant pH yielded linear relationships. Notably, *k*_obsd_ can be expressed as Equation (2), where *k*_o_ is a constant that depends only on pH.
(2)

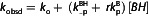




**Figure 2:**
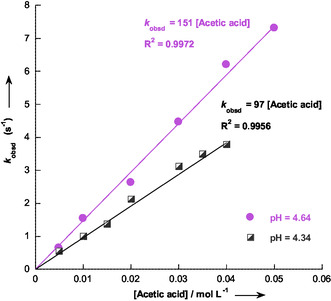
Effect of buffer concentration and pH on the observed rate constant, *k*_obsd_, for the protonation of 1‐hydroxy‐2,2‐dinitroethanide **C‐1** in acetic acid buffer in aqueous solution at 25 °C, with an ionic strength of 0.1 mol L^−1^ KCl.

For each buffer, equilibrium according to Scheme 4 was investigated by systematically varying the buffer ratio *r* = [*B*]/[*BH*], thereby covering a range of pH values. **Figure** **2** presents the results obtained for acetic acid buffers, while additional data for other buffers are provided in (Figure S2–S7, Supporting Information). From the slopes of these linear plots, the individual rate constants 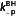 and $k_{p}^{B}$ were determined at two different buffer ratios, *r *= [*B*]/[*BH*]. The resulting rate constants are presented in **Table** **2**.

**Table 2:** Second‐order rate constants for the proton transfer reactions of carbanion C‐1with various carboxylic buffers in aqueous solution, 25 °C, and *I* = 0.1 mol L^−1^ KCl.

Entry	Buffer (acid species)	p*K*_a_ a)	$k_{p}^{B}$ [mol^−1^ L s^−1^]b)	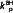 [mol^−1^ L s^−1^]b)
(a)	Cacodylic acid	6.15	832	6.60
(b)	Succinate ion	5.60	525	13.80
(c)	Acetic acid	4.64	107	43.65
(d)	Formic acid	3.60	31.62	100
(e)	Methoxyacetic acid	3.45	22.91	129
(f)	Chloroacetic acid	2.71	9.77	162
(g)	Cyanoacetic acid	2.37	8.13	309

a) p*K*_a_ values of carboxylic acid measured in aqueous solution at 25 °C and *I* = 0.1 mol L^−1^ KCl were taken from ref.[36].

b) These rate constants were derived from the slopes of *k*_obsd_ versus acid species plots (see Figure 2 and Supporting Information for details).

## 3 Discussion

### 3.1 Understanding the Protonation Pathway of 1‐Hydroxy‐2,2‐Dinitroethanide C‐1 in HCl Solutions: Acidity of the Nitronic Acid C‐1H

**Figure 3:**
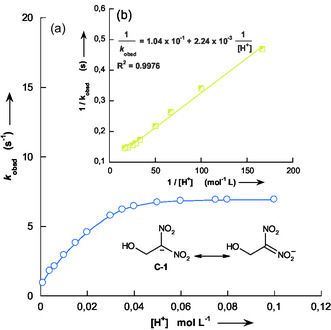
a) Dependence of the pseudo‐first‐order rate constants (*k*_obsd_) on the H_3_O^+^ concentration, for the protonation of 1‐hydroxy‐2,2‐dinitroethanide **C‐1** with hydrochloric acid solutions in water at 25 °C and *I* = 0.1 mol L^−1^ maintained with KCl. b) Inversion plot according to Equation (4) for protonation of the carbanion **C‐1** in the pH range 1–3 in water.

Under the experimental conditions employed, the kinetic results given in Table 1 revealed that the plot of *k*_obsd_ versus [H_3_O^+^] exhibits a characteristic curvature, reaching a plateau at higher acid concentrations (**Figure** **3**a). Similar behavior has been reported in the literature.^[^ ^10^, ^12^, ^14^ ^]^ A typical example is the protonation of the carbanion **C‐2** ^[^ ^14^ ^]^ and **C‐3**,^[^ ^12^ ^]^ formed from 4‐X‐substituted picrylacetophenones **2** and (4‐nitrophenyl)nitromethane **3**, respectively, in a 50% H_2_O–50% DMSO mixture. In this case, it has been proposed that the carbanion undergoes immediate protonation at its nitro group, which is one of the hypotheses put forward to explain the irregular kinetic behavior observed.^[^ ^10^, ^12^, ^14^ ^]^ This behavior has been attributed to a competition between protonation at the carbon and oxygen sites, leading to complex kinetic profiles.


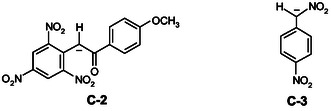


**Scheme 5:**
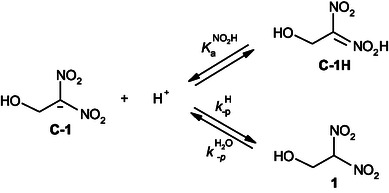
Proposed mechanistic pathway for the proton‐transfer reactions of 1‐hydroxy‐2,2‐dinitroethanide **C‐1** with hydrochloric acid in aqueous solution at 25 °C and *I* = 0.1 mol L^−1^ KCl.

In our study, it seems reasonable to anticipate that the experimental data reported here is explained by the transient intermediate corresponding to a nitronic acid species **C‐1H**, formed via protonation at the NO_2_ group of carbanion **C‐1**, as illustrated in **Scheme** **5**.

The mechanism implies an initial fast protonation of carbanion **C‐1** to generate an intermediate species **C‐1H**, followed by a slower conversion of this intermediate into the final acid **1**. The presence of a plateau at high [H_3_O^+^] concentrations suggests that, under these conditions, the overall reaction rate is governed by the decomposition of **C‐1H** rather than the initial protonation step. This behavior can be modeled using a saturation‐type Equation (3), where $K_{a}^{{\text{NO}}_{2}H}$ is the acidity constant of the nitronic acid functionality and 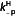 and 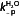 are the rate constants referring to the reprotonation of **C‐1** by H_3_O^+^ and the solvent, respectively.
(3)

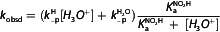




If the contribution of the solvent (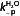) in Equation (3) is negligible, the equation simplifies to:

(4) $$\frac{1}{k_{\text{obsd}}}=\frac{1}{k_{\text{‐p}}^{H}\;[H_{3}O^{+}]}+\;\frac{1}{k_{\text{‐p}}^{H}\;K_{a}^{{\text{NO}}_{2}H}}$$

In accordance with Equation (4), the plot of $\frac{1}{k_{\text{obsd}}}$ versus $\frac{1}{\left[H_{3}O^{+}\right]}$ exhibits a linear relationship (Figure 3b), indicating that the reaction follows a well‐defined kinetic model. By performing a least‐squares regression on the experimental data, we can accurately determine the kinetic parameters governing the protonation process. Specifically, the slope of the linear fit corresponds to the rate constant $\frac{1}{k_{\text{‐p}}^{H}\;}$, while the intercept provides the value of $\frac{1}{k_{\text{‐p}}^{H}\;K_{a}^{{\text{NO}}_{2}H}}$. From our experimental data, we obtained $\frac{1}{k_{\text{‐p}}^{H}\;}$ = 2.24 × 10^−3^ mol L^−1^ s^−1^ and $\frac{1}{k_{\text{‐p}}^{H}\;K_{a}^{{\text{NO}}_{2}H}}$ = 1.04 × 10^−1^ s^−1^. These values allow for the calculation of the acidity constant $pK_{a}^{{\text{NO}}_{2}H}$ of **C‐1H**, which quantifies the extent of the protonation process at the oxygenated site of the carbanion **C‐1** under the given conditions, yielding a value of 1.67.

The protonation of nitronate anions under acidic conditions has been extensively investigated by several research groups.^[^ ^7^, ^10^, ^11^, ^12^, ^14^, ^15^, ^16^ ^]^ Kinetic studies have allowed for the determination of the acidity constants of different protonation sites while identifying the electronic and steric effects influencing selectivity, thereby enhancing the understanding of the behavior of nitronate anions in acidic conditions. **Table** **3** reflects this situation by comparing the acidity constants of several carbon acid structures measured in H_2_O and in mixed DMSO–H_2_O solvents at 25 °C.

**Table 3:** Comparison of acidity constants of the nitronic acid functionality ($pK_{a}^{{\text{NO}}_{2}H}$) of some representative nitronic acid structures in H_2_O and in mixed DMSO–H_2_O solvents at 25 °C.

Nitronic acid structures	Solvent	$pK_{a}^{{\text{NO}}_{2}H}$	Reference
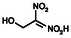 **C‐1H**	H_2_O	1.67	This work
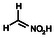 **C‐4H**	H_2_O	3.25	[11]
	10% H_2_O–90% DMSO	8.65	[7]
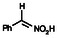 **C‐5H**	H_2_O	3.64	[7]
	10% H_2_O–90% DMSO	7.73	
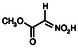 **C‐6H**	H_2_O	0.98	[11]
	50% H_2_O–50% DMSO	1.43	
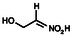 **C‐7H**	H_2_O	3.44	[15]
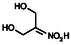 **C‐8H**	H_2_O	3.60	[15]
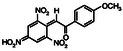 **C‐2H**	50% H_2_O–50% DMSO	3.17	[14]
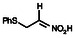 **C‐9H**	10% H_2_O–90% DMSO	8.55	[16]
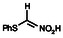 **C‐10H**	10% H_2_O–90% DMSO	6.79	[17]]

### 3.2 Thermodynamic Acidity of 1‐Hydroxy‐2,2‐Dinitroethane 1 in Aqueous Solution: C‐Protonation and Nitronic Acid Equilibria

**Figure 4:**
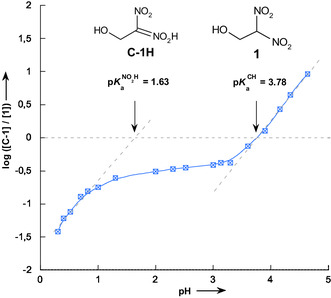
Variation of the ratio of ionized **C‐1** to unionized 1‐hydroxy‐2,2‐dinitroethane **1** as a function of pH in aqueous solution at 25 °C, *I* = 0.1 mol L^−1^ KCl.

The pH dependence of equilibria **I** and **II** were investigated using dilute hydrochloric acid solutions as well as various methoxyacetic and acetic acid buffer solutions. The equilibrium absorbance variations at the absorption maximum of the carbanion **C‐1** (*λ*max = 367 nm) were monitored as a function of pH, allowing for the construction of a log [**C‐1**]/[**1**] versus pH plot. As illustrated in **Figure** **4**, this plot reveals the existence of two distinct thermodynamic processes, indicating multiple equilibria governing the protonation states of the system.


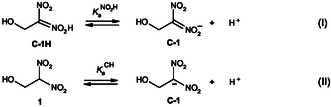


Based on previous findings, upon mixing **C‐1** with a strongly acidic solution, the system does not only undergo direct conversion of **C‐1** into **1**, but also experiences rapid oxygen protonation, forming the nitronic acid intermediate in a pre‐equilibrium before further partitioning, as depicted in Scheme 5. The first segment, corresponding to the pH range 0.30 < pH < 1.00, can be attributed to the protonation of **C‐1** into **C‐1H**, as defined by Equation (5). The second segment, spanning 3.30 < pH < 4.64, represents the conversion of **C‐1** to **1**, as described by Equation (6).

(5) $$\log[C\text{-}1H]/[1]=-1.63+0.96\;\text{pH}\;\;(R^{2}=0.9720)$$

(6) $$\log[C\text{-}1]/[1]=-3.78+1.02\;\text{pH}\;\;\;\;\;\;(R^{2}=0.9959)$$

Interestingly, both linear fits exhibit nearly identical slopes of ≈1.00, suggesting that Equations

(5) and (6) can be reliably used to determine the acidity constants of the two equilibria **I** and **II**. Accordingly, analysis of Equation (5) yields $pK_{a}^{{\text{NO}}_{2}H}$ = 1.63, while Equation (6) gives $pK_{a}^{\text{CH}}$ = 3.78. Notably, the $pK_{a}^{{\text{NO}}_{2}H}$ = 1.63 value measured for the nitronic acid **C‐1H** is in good agreement with the kinetically determined value obtained in this work ($pK_{a}^{{\text{NO}}_{2}H}$ = 1.67), reinforcing the consistency of the experimental approach and providing a more comprehensive understanding of the protonation behavior of 1‐hydroxy‐2,2‐dinitroethane **1**.

**Figure 5:**
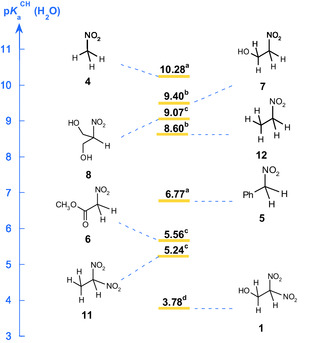
Comparison of $pK_{a}^{\text{CH}}$ values for 1‐hydroxy‐2,2‐dinitroethane **1** and some representative nitroalkane structures in water at 25 °C. ^a^From ref. [7]. ^b^From ref. [15]. ^c^From ref. [11]. ^d^This work.

**Figure** **5** presents a comparative analysis of the acidity of 1‐hydroxy‐2,2‐dinitroethane **1** and selected NO_2_‐substituted carbon acids in water at 25 °C.^[^ ^7^, ^11^, ^15^ ^]^ The data reveal substantial differences in acidity among these compounds, underscoring the profound influence of structural and electronic effects on their proton dissociation equilibria. Notably, 1‐hydroxy‐2,2‐dinitroethane **1** exhibits a remarkably low $pK_{a}^{\text{CH}}$, significantly surpassing the acidity of related nitroalkane derivatives. Specifically, $pK_{a}^{\text{CH}}$ is 1.78 units lower than that of methyl nitroacetate **6**, 2.99 units lower than that of nitrobenzyl **5**, and an astonishing 6.50 units lower than that of nitromethane **4** (Figure 5). This pronounced acidity can be directly attributed to the presence of a second NO_2_ group, which dramatically enhances the stabilization of the conjugate base via a combination of strong inductive withdrawal and extensive resonance delocalization. Consistent with prior reports, the introduction of a second NO_2_ group dramatically increases acidity: the transformation of 2‐nitroethanol **7** ($pK_{a}^{\text{CH}}$ = 9.40) into 1‐hydroxy‐2,2‐dinitroethane **1** results in a striking $\Delta pK_{a}^{\text{CH}}$ of 5.62 units.

The impact of polynitro substitution on carbon acidity is well‐documented, and our findings align closely with the $pK_{a}^{\text{CH}}$ **= **5.24 reported by Bernasconi for 1,1‐dinitroethane **11**,^[^ ^11^ ^]^ further corroborating the critical role of multiple NO_2_ groups in modulating acidity.

Interestingly, compound **1** is significantly more acidic than 1,1‐dinitroethane **11** ($pK_{a}^{\text{CH}}$ = 5.24), despite both molecules bearing two nitro groups on the same carbon. At first impression, this observation may seem inconsistent with Bernasconi's trend reported for mononitroalkanes, where the introduction of a CH_2_OH group leads to an increase in $pK_{a}^{\text{CH}}$ (e.g., nitroethane **12**, $pK_{a}^{\text{CH}}$ = 8.60^[^ ^15^ ^]^ vs. 2‐nitroethanol **7**, $pK_{a}^{\text{CH}}$ = 9.40^[^ ^15^ ^]^). However, the discrepancy is resolved by considering the nature of the substituent adjacent to the acidic center. In compound **1**, the electron‐withdrawing CH_2_OH group replaces the methyl group found in 1,1‐dinitroethane **11**.^[^ ^11^ ^]^ This hydroxymethyl substituent exerts a stronger inductive effect, significantly increasing the acidity by stabilizing the conjugate base. Moreover, potential intramolecular hydrogen bonding between the OH and a neighboring nitro oxygen may further enhance this stabilization. These combined electronic and structural effects rationalize the enhanced acidity of compound **1**, beyond what is observed for its methylated analog.

### 3.3 Intrinsic Reactivity of 1‐Hydroxy‐2,2‐Dinitroethane 1 in Water

**Figure 6:**
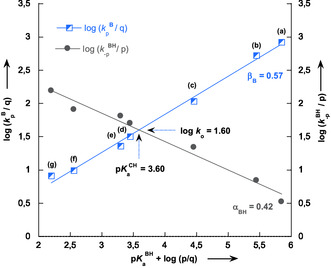
Statistically corrected Brönsted plots for the proton‐transfer reactions of 1‐hydroxy‐2,2‐dinitroethanide **C‐1** in carboxylic acid buffer solutions in water at 25 °C, *I* = 0.1 mol L^−1^ KCl. The identity of the points is given in Table 2. The $pK_{a}^{\text{BH}}$values were taken from ref. [36]

The rate constants 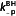 and $k_{p}^{B}$ (Table 2) related to the protonation of **C‐1** by carboxylic acids and the deprotonation of **1** by their conjugate bases follow well‐defined linear Brönsted relationships,^[^ ^17^, ^18^, ^19^, ^20^, ^21^ ^]^ with high correlation coefficients (*R* ^2^ > 0.98). These correlations, illustrated in **Figure** **6**, are quantitatively expressed by the following equations.

(7) $$\log\;k_{p}^{B}=-0.45+0.57\;pK_{a}^{\text{BH}}\;(R^{2}=0.9967)$$

(8) $$\log\;k_{-p}^{\text{BH}}=3.14-0.42\;pK_{a}^{\text{BH}}\;(R^{2}=0.9879)$$

An intriguing feature of Figure 6 is the intersection of the two lines at a point that corresponds to the acidity constant $pK_{a}^{\text{CH}}$ of **1**, which is equal to 3.60. Notably, this value is in remarkable agreement with the one determined according to Equation (6) ($pK_{a}^{\text{CH}}$ = 3.78). On the other hand, the Brönsted coefficients *β*_B_ and *α*_BH_, determined as 0.57 and 0.42, respectively, fall within the typical range of 0.50 ± 0.10, commonly observed for these parameters in the deprotonation of carbon acids by various catalysts.^[^ ^10^, ^12^, ^14^, ^22^, ^23^ ^]^ For example, *β*_B_ = 0.55 and *α*_BH_ = 0.45 have been reported by Moutiers and coworkers for deprotonation of (4‐nitrophenyl)nitromethane by various phenoxide ions in 50% DMSO–50% Water v/v.^[^ ^12^ ^]^

It is worth noting that these linear correlations can be used to estimate the intrinsic reactivity of 1‐hydroxy‐2,2‐dinitroethane **1**. In fact, by applying Marcus's approach,^[^ ^24^ ^]^ the intrinsic rate constant, *k*_o_ = $k_{p}^{B}$/*q*, can be determined when $pK_{a}^{\text{CH}}$–$pK_{a}^{\text{BH}}$–log (*p*/*q*) = 0. Using the Brönsted plots shown in Figure 6, the log *k*_o_ value for nitroalkane **1** was found to be log *k*_o_ = 1.60.

**Scheme 6:**
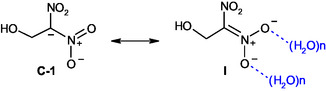
Resonance structures **C‐1** and **I** illustrating the electron delocalization in the conjugate base of 1‐hydroxy‐2,2‐dinitroethane **1**.

The measured log *k*_o_ value of 1.60 for 1‐hydroxy‐2,2‐dinitroethane **1** is remarkably low compared to *α*‐dinitriles of the general formula RR′C(CN)_2_, which typically exhibit log *k*_o_ values around 7.00.^[^ ^8^ ^]^ This pronounced difference highlights the dominant role of charge delocalization via the nitro groups in stabilizing the resulting carbanion formed upon deprotonation at the C‐1 position. The conjugate base of **1** benefits from extensive resonance stabilization, as illustrated by resonance structures **C‐1** and **I** (see **Scheme** **6**). In structure **C‐1**, the negative charge remains localized at the *α*‐carbon, whereas in structure **I**, it is delocalized onto one of the nitro groups via a resonance pathway that generates a nitronate‐like structure. This highly efficient delocalization is far more stabilizing than the charge distribution possible in dinitriles, where the negative charge remains primarily localized on the central carbon atom due to the lower *π*‐accepting ability of cyano groups compared to nitro groups. Moreover, this strong delocalization over the nitro groups also accounts for the observed tendency toward O‐protonation, which occurs because the substantial electron density located on the oxygen atoms of the nitro groups makes them favorable sites for protonation, as opposed to the *α*‐carbon.

The log *k*_o_ value of 1.60 determined in this work for 1‐hydroxy‐2,2‐dinitroethane **1**, along with the previously reported value of 1.00 for 1,1‐dinitroethane **11** by Bernasconi,^[^ ^11^ ^]^ related to deprotonation by carboxylate ions in aqueous solution at 25 °C, clearly demonstrate that these two dinitroalkanes exhibit significantly higher intrinsic proton transfer rates (log *k*_o_) compared to their mononitro counterparts, such as 2‐nitroethanol **7** (log *k*_o_ = −0.90),^[^ ^15^ ^]^ 2‐nitro‐1,3‐propanediol **8** (log *k*_o_ = −1.09),^[^ ^11^ ^]^ and the nitrobenzyl derivative **5** (log *k*_o_ = −2.10),^[^ ^7^ ^]^ all measured under identical conditions. These observations clearly highlight that the substantial increase in log *k*_o_ values observed for dinitroalkanes compared to their mononitro analogs stems from the enhanced stabilization of the carbanionic conjugate base by the second electron‐withdrawing NO_2_ group. This group reinforces both inductive and resonance effects, thereby lowering the activation barrier for proton transfer and substantially increasing the intrinsic rate constant (log *k*_o_).

### 3.4 Proton‐Transfer Correlations ‐ Exploring the Relationship Between $pK_{a}^{{\text{NO}}_{2}H}$, $pK_{a}^{\text{CH}}$ and p*K*_N_

Building on the pioneering work of Bernasconi and coworkers, who attempted to correlate intrinsic reactivity (log *k*_0_) with the acidity constants ($pK_{a}^{\text{CH}}$) of various nitroalkanes,^[^ ^11^ ^]^ we sought to elucidate possible interrelationships between three fundamental descriptors: the acidity constant of the nitronic acid ($pK_{a}^{{\text{NO}}_{2}H}$), the acidity constant of the parent nitroalkane ($pK_{a}^{\text{CH}}$), and the tautomeric equilibrium constant, defined as p*K*_N_ = $pK_{a}^{\text{CH}}$−$pK_{a}^{{\text{NO}}_{2}H}$. This analysis aims to reveal how these distinct acidity measures reflect the proton‐transfer behavior of nitroalkanes in aqueous solution and whether they are mechanistically or thermodynamically interrelated.

**Figure 7:**
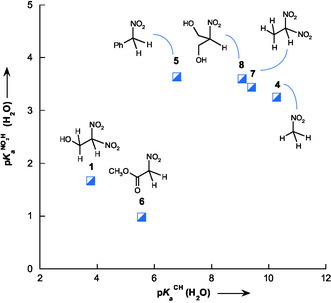
Plot of the nitronic acidity constant ($pK_{a}^{{\text{NO}}_{2}H}$) versus the acidity constant ($pK_{a}^{\text{CH}}$) for selected nitroalkanes **1** and **4–8** in water at 25 °C.

**Scheme 7:**
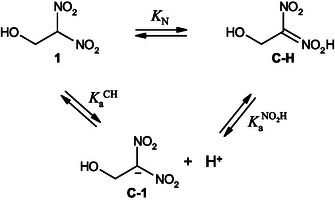
Tautomeric equilibrium: Proton transfer and aci C—H species formation 1‐hydroxy‐2,2‐dinitroethane **1**.

Despite a systematic comparison of $pK_{a}^{\text{CH}}$ and $pK_{a}^{{\text{NO}}_{2}H}$ for a representative series of nitroalkanes **1** and **4–8** (Table 3, Figure 5), no significant linear correlation emerged (**Figure** **7**). This result suggests that these two acidity constants are governed by fundamentally different electronic and structural factors. While $pK_{a}^{\text{CH}}$ measures the ease of deprotonation at the *α*‐carbon, closely linked to the stability of the resulting carbanion, $pK_{a}^{{\text{NO}}_{2}H}$reflects the relative thermodynamic stability of the nitronic (aci) tautomer formed through an intramolecular proton shift to the nitro group. The decoupling of these two acidity scales underscores the coexistence of distinct deprotonation pathways in nitroalkane systems: direct ionization of the *α*‐C—H bond versus formation of the nitronic acid tautomer. To provide a unified framework, we introduced the tautomeric equilibrium constant, p*K*_N_, as a more informative descriptor. This parameter captures the thermodynamic balance between the neutral nitroalkane and its aci‐form, offering a direct measure of the compound's tendency to tautomerize. A lower p*K*_N_ value indicates a greater propensity for aci‐tautomer formation. This concept is illustrated in **Scheme** **7** for nitroalkane **1**.

**Figure 8:**
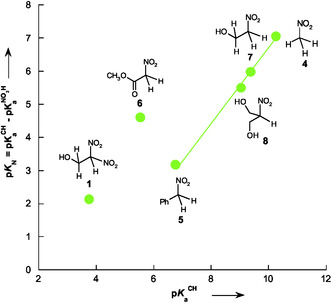
Plot of tautomeric equilibrium constant (p*K*_N_) versus the acidity constant ($pK_{a}^{\text{CH}}$) for selected nitroalkanes**1** and **4–8** in water at 25 °C.

When p*K*_N_ was plotted against $pK_{a}^{\text{CH}}$ (**Figure** **8**), a remarkably linear correlation was observed for nitroalkanes **4**–**8**, with a regression described by Equation (9) (*R* ^2^ = 0.9981).

(9) $$pK_{N}=-4.36\;+\;1.10\;pK_{a}^{\text{CH}}$$

Notably, compounds **1** and **6** deviate from this trend. For compound **1**, this deviation may be attributed to its unusually high intrinsic proton transfer rate (log *k*_o_ = 1.60), measured in this study. This reactivity is significantly greater than that of the other acids considered in Figure 8, which exhibit log *k*_o_ values between −2.10 and −0.90. A log *k*_o_ this high could be explained by better stabilization of the transition state or by specific interactions with the solvent, which increase the proton transfer rate beyond predictions based solely on the electronic structure. Although no experimental log *k*_o_ value is available for compound **6**, its position in the plot similarly suggests that additional structural or environmental factors, such as intramolecular interactions or conformational effects, could influence its acidity and tautomeric equilibrium.

Despite these deviations, the correlation in Figure 8 remains a reliable tool for estimating $pK_{a}^{{\text{NO}}_{2}H}$ values of structurally related nitroalkanes, particularly in cases where experimental p*K*_N_ data are not accessible. The inclusion of compounds **1** and **6** in the regression analysis still yields a satisfactory correlation (*R* ^2^ = 0.9120), as shown in Figure S8, Supporting Information. However, to maintain a robust and generalizable model, Equation (9) was derived from the set of compounds exhibiting consistent and representative behavior within the series.

Based on the considerations discussed above regarding compounds **1** and **6**, Equation (9) can still be considered a relevant tool for estimating the nitronic acid acidity ($pK_{a}^{{\text{NO}}_{2}H}$) of nitroalkanes for which direct experimental data are unavailable. This approach is particularly useful when the experimental determination of the tautomeric equilibrium constant (p*K*_N_) is challenging. We applied this model to estimate the $pK_{a}^{{\text{NO}}_{2}H}$ values for four nitroalkanes **11** and **13**–**15**, for which only $pK_{a}^{\text{CH}}$ values in water are reported.^[^ ^16^, ^17^, ^18^ ^]^ The results, summarized in **Table** **4**, demonstrate that the predicted $pK_{a}^{{\text{NO}}_{2}H}$ values are in good agreement with Bernasconi's hypothesis, suggesting that the $pK_{a}^{{\text{NO}}_{2}H}$ values for PhSCH_2_NO_2_ **13** and PhCH_2_CH_2_NO_2_ **14** are expected to be below 4.0 in this solvent.^[^ ^16^ ^]^

**Table 4:** Prediction of the tautomeric equilibrium constant (p*K*_N_) and acidity constants of nitronic acid ($pK_{a}^{{\text{NO}}_{2}H}$) for selected nitroalkanes **11** and **13–15** in water at 25 °C based on the correlation in Equation (9).

Nitroalkane structures	$pK_{a}^{\text{CH}}$	p*K*_N_ d)	$pK_{a}^{{\text{NO}}_{2}H}$ d)
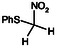 **13**	6.67a)	2.98	3.69 <4e)
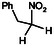 **14**	8.55a)	5.04	3.51 <4e)
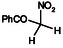 **15**	4.67b)	0.78	3.89
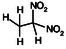 **11**	5.24c)	1.40	3.84

a) From ref. [16].

b) From ref. [18].

c) From ref. [11].

d) Values determined in this work using Equation (9).

e) Estimated value reported by Bernasconi.^[^ ^16^ ^]^

While nucleophilicity parameters (*N*)^[^ ^25^ ^]^ versus acidity constants ($pK_{a}^{\text{CH}}$) correlations are now well established for C‐deprotonated species, to the best of our knowledge, no systematic study has yet addressed the acidity of O‐deprotonated nitronates (i.e., nitronic acids, $pK_{a}^{{\text{NO}}_{2}H}$) nor the corresponding tautomeric constants (p*K*_N_). This important gap in the structure–acidity–reactivity landscape of nitroalkanes has remained largely unexplored, despite the relevance of nitronate anions in both physical organic chemistry and synthetic applications.

In our recent investigations, we reported predictive relationships between the experimental nucleophilicity parameters (*N*) and acidity constants ($pK_{a}^{\text{CH}}$) of nitroalkyl carbanions in methanol,^[^ ^26^ ^]^ in combination with the theoretical nucleophilicity index (*ω* ^−1^) based on Parr's model.^[^ ^27^ ^]^ Similar studies by Mayr and coworkers in DMSO and water^[^ ^28^ ^]^ have highlighted the role of the NO_2_ group in modulating carbanion reactivity and stabilization.

Building upon this dual experimental–theoretical approach, we plan to extend our current study by including a set of calculated proton affinities (PA)^[^ ^29^ ^]^ at the carbon and oxygen atoms of nitronate anions, as well as the theoretical nucleophilicity indices (*ω* ^−1^) obtained from density functional theory calculations. These descriptors will be combined with the experimentally determined nucleophilicity parameters (*N*) in order to explore quantitative relationships with the three acidity‐related constants: the *α*‐C—H acidity **(** $pK_{a}^{\text{CH}}$ **)**, the nitronic acid acidity ($pK_{a}^{{\text{NO}}_{2}H}$), and the tautomeric equilibrium constant (*pK*_N_).

Our objective is to identify and rationalize structure–property relationships that govern the distribution of basicity (carbon vs. oxygen), the position of tautomeric equilibria, and the nucleophilic reactivity of nitroalkane‐derived species. Such a multidimensional correlation, bridging experimental and theoretical descriptors, is expected to provide a predictive framework for interpreting and anticipating the behavior of structurally related nitro compounds in aqueous solution.

## 4 Conclusion

This study comprehensively investigates the reversible protonation of 1‐hydroxy‐2,2‐dinitroethanide **C‐1** by hydrochloric acid and various carboxylic acid buffer solutions in water at 25 °C. The reaction mechanism was elucidated, leading to the determination of acidity constants for both O‐protonation ($pK_{a}^{{\text{NO}}_{2}H}$ = 1.67) and C‐protonation ($pK_{a}^{\text{CH}}$ = 3.78). Notably, the introduction of a second NO_2_ group into OHCH_2_CH_2_NO_2_ to form 1‐hydroxy‐2,2‐dinitroethane **1** resulted in a significant increase in the acidity of both the C—H bond in acid **1** and the O—H bond in nitronic acid **C‐1H**, with increases of ≈5.62 and 1.77 p*K*_a_ units, respectively. These findings reinforce the idea that delocalization through the NO_2_ groups plays a crucial role in stabilizing the nitronate anion in nitroalkanes. Based on the solid framework established by correlation (9), we have developed a predictive model to estimate the acid dissociation constants of nitronic acids ($pK_{a}^{{\text{NO}}_{2}H}$) derived from four nitroalkanes (PhCH_2_NO_2_ **13**, PhCH_2_CH_2_NO_2_ **14**, CH_3_CH(NO_2_)_2_ **15**, and PhCOCH_2_NO_2_ **16**) in water, which are not directly accessible experimentally. These findings emphasize the sensitivity of tautomeric equilibria to subtle molecular and environmental variations and suggest that future studies exploring a broader structural space will be essential to refine and generalize this predictive relationship.

## 5 Experimental Section

5.1

5.1.1

##### Materials

All secondary carboxylic acids, including cacodylic acid, succinate ion, acetic acid, formic acid, methoxyacetic acid, chloroacetic acid, and cyanoacetic acid, were commercially obtained from Aldrich and used without further purification.

1‐hydroxy‐2,2‐dinitroethane 1 was synthesized following a procedure similar to that previously described for related aliphatic nitroalkanes.^[^ ^15^ ^]^

##### Kinetic Measurements

Kinetic measurements of the proton transfer reactions were conducted using a Shimadzu UV–vis spectrophotometer (Model 1650) and a BioLogic Stopped‐Flow spectrophotometer (Model SFM‐X00/Q). Temperature control was ensured using a thermoelectrically regulated cell holder (Model TCC‐240A), maintaining a stable temperature of 25.0 ± 0.1 °C. Pseudo‐first‐order rate constants *k*_obsd_ were calculated using Equation (10),^[^ ^30^, ^31^, ^32^ ^]^ where *A*_∞_ refers to the absorbance of 1‐hydroxy‐2,2‐dinitroethanide **C‐1** at the completion of the reaction, *A*_o_ refers to the absorbance at zero time, and *A*_t_ refers to the absorbance at time *t*. In a given experiment, the rates were found to be reproducible to ± 3–5%. Correlation coefficients of the linear regressions were usually higher than 0.97. The variations of the observed rate constants *k*_obsd_ as a function of the concentrations of the carboxylic acids are shown in (Figure S2–S7, Supporting Information).

(10) $$ln\;\left(A_{\infty }-A_{t}\right)\;=\;-k_{\text{obsd}}t\;+\;ln\;\left(A_{\infty }-A_{o}\right)$$

##### Acidity Measurements

The thermodynamic acidity constant of **1** was determined in aqueous solution at 25 °C by spectrophotometrically monitoring the equilibrium absorbance variations at the absorption maxima of the corresponding carbanion **C‐1** as a function of pH. Various carboxylic acid (*BH*) buffer solutions with [*B*]/[*BH*] ratios ranging from 4/1 to 1/1 were used, while maintaining a constant ionic strength of 0.1 mol L^−1^ KCl in aqueous solution. The $pK_{a}^{\text{CH}}$ value of **1** was calculated using Equation (11),^[^ ^33^, ^34^, ^35^ ^]^ where *A* represents the equilibrium absorbance, *A*_C‐1_ is the absorbance of **C‐1**, and *A*_1_ corresponds to the absorbance of the carbon acid **1**.
(11)

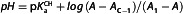




## In Memoriam: Professor François Terrier

In memory of Professor François Terrier, a distinguished chemist and exceptional educator, who passed away on July 26, 2024. His scientific rigor, intellectual generosity, and constant availability left a lasting impression on all who had the privilege of working with him.

Renowned for his pioneering work in physical organic chemistry, Professor François Terrier was a guiding figure at the beginning of my research career. My early work, dedicated to the study of the ionization of carbon acids, was directly inspired by his approaches. The present article, which continues this foundational theme, stands as a modest scientific tribute to his memory.

I had the honor of cosupervising several Ph.D. theses alongside Professor Terrier, which led to the training of researchers who are now well established in their respective fields: Dr. S. Lakhdar (CNRS Research Scientist in Toulouse), Dr. N. El Guesmi (assistant professor at the Faculty of Sciences of Monastir), Dr. W. Gabsi (lecturer in Saudi Arabia), Dr. N. Dharhi (professional in the banking sector in France), and Dr. A. Echaieb (Director of Research and Development in the FMCG sector, Sweden).

In continuation of the research themes initiated by Professor Terrier, I have also had the privilege of supervising several doctoral students whose work directly follows this scientific lineage. This dynamic is carried forward today by young researchers holding positions as temporary teaching and research assistants (ATER) or postdoctoral fellows in both France and Tunisia. Among them are FaouziMahdhaoui (University of Lorraine), HanenRaissi (University of Picardie Jules Verne), Salma Souissi (Aix‐Marseille University), FerielNecibi (University of Paris‐EstCréteil), HajerAyachi (Faculty of Sciences of Monastir), TakwaSlama (Higher Institute of Biotechnology of Monastir), and OnsAmamou (Sorbonne University).

Through their dedication and the relevance of their research, these young scientists are faithfully and confidently carrying forward the scientific legacy of Professor Terrier. We express our deepest gratitude to him and extend our sincere condolences to his family, his loved ones, and the entire scientific community that he so deeply inspired.

## Conflict of Interest

The authors declare no conflict of interest.

## Author Contributions

**Rania Khaldi**: Conducted the experimental work with the assistance of Taoufik Boubaker. Responsible for conceptualization, investigation, and methodology. **Amel Hedhli**: Contributed to investigation and methodology. **Taoufik Boubaker**: Responsible for writing original draft, supervision, and validation.

## Supporting information

Supplementary Material

## Data Availability

The data that support the findings of this study are available in the supplementary material of this article.

## References

[1] A. Y. Sukhorukov , Molecules 2023, 28, 686.36677743

[2] L. Schubert , P. Langner , D. Ehrenberg , V. A. Lorenz‐Fonfria , J. Heberle , J. Chem. Phys. 2022, 156, 204201. 35649857 10.1063/5.0088526

[3] G. Baccolini , G. Bartoli , M. Bosco , R. Dalpozzo , J. Chem. Soc., Perkin Trans. 2 1984, 363.

[4] G. Bartoli , M. Bosco , R. Dalpozzo , P. Sgarabotto , J. Chem. Soc., Perkin Trans. 2 1982, 929.

[5] P. G. Farrell , F. Terrier , H.‐Q. Xie , T. Boubaker , J. Org. Chem. 1990, 55, 2546.

[6] F. Terrier , H.‐Q. Xie , J. Lelievre , T. Boubaker , P. G. Farrell , J. Chem. Soc., Perkin Trans. 2 1990, 11, 1899.

[7] C. F. Bernasconi , D. A. V. Kliner , A. S. Mullin , J. X. Ni , J. Org. Chem. 1988, 53, 3342.

[8] C. F. Bernasconi , Tetrahedron 1985, 41, 3219.

[9] P. Fogel , P. G. Farrell , J. Lelievre , A. P. Chatrousse , F. Terrier , J. Chem. Soc., Perkin Trans. 2 1985, 711.

[10] G. Moutiers , B. El Fahid , A.‐G. Collot , F. Terrier , J. Chem. Soc., Perkin Trans. 2 1996, 49.

[11] C. F. Bernasconi , M. P‐Lorenzo , S. D. Brown , J. Org. Chem. 2007, 72, 4416. 17500569 10.1021/jo070372r

[12] G. Moutiers , V. Thuet , F. Terrier , J. Chem. Soc., Perkin Trans. 2 1997, 1479.

[13] (a) K. Ando , Y. Shimazu , N. Seki , H. Yamataka . J. Org. Chem. 2011, 76, 3937. 21486082 10.1021/jo200383f

[14] G. Moutiers , B. El Fahid , R. Goumont , A. P. Chatrousse , F. Terrier , J. Org. Chem. 1996, 61, 1978.

[15] C. F. Bernasconi , M. Panda , M. W. Stronach , J. Am. Chem. Soc. 1995, 117, 9206.

[16] C. F. Bernasconi , K. W. Kittredge , J. Org. Chem. 1998, 63, 1944.

[17] C. F. Bernasconi , A. Kanavarioti , J. Org. Chem. 1979, 44, 4829.

[18] C. F. Bernasconi , R. L. Montañez , J. Org. Chem. 1997, 62, 8162. 11671926 10.1021/jo971259b

[19] F. G. Bordwell , J. E. Bartmess , J. A. Hautala , J. Org. Chem. 1978, 43, 3107.

[20] R. A. Marcus , J. Am. Chem. Soc. 1969, 91, 7224.

[21] F. Terrier , H. Q. Xie , P. G. Farrell , J. Org. Chem. 1990, 55, 2610.

[22] C. F. Bernasconi , P. Paschalis , J. Am. Chem. Soc. 1986, 108, 2969.

[23] C. F. Bernasconi , D. Wiersema , M. W. Stronach , J. Org. Chem. 1993, 58, 217.

[24] R. A. Marcus , J. Phys. Chem. 1968, 72, 891.

[25] H. Mayr , M. Patz , Angew. Chem. Int. Ed. Engl. 1994, 33, 938.

[26] F. Necibi , S. B. Salah , J.‐C. Hierso , P. Fleurat‐Lessard , S. Ayachi , T. Boubaker , Chem. Select 2023, 8, e202203590.

[27] R. G. Parr , L. V. Szentpaly , S. Liu , J. Am. Chem. Soc. 1999, 121, 1922.

[28] B. Thorsten , L. Tadeusz , H. Mayr , J. Org. Chem. 2004, 69, 7565. 15497983 10.1021/jo048773j

[29] W. Danikiewicz , Int. J. Mass Spectrom. 2009, 285, 86.

[30] F. Terrier , T. Boubaker , L. Xiao , P. G. Farrell , J. Org. Chem. 1992, 57, 3924.

[31] F. Terrier , E. Kizilian , R. Goumout , N. Faucher , C. Wakselman , J. Am. Chem. Soc. 1998, 120, 9496.

[32] G. Moutiers , E. Le Guével , L. Villien , F. Terrier , J. Chem. Soc., Perkin Trans. 2 1997, 7.

[33] T. Boubaker , A. P. Chatrousse , F. Terrier , B. Tangour , J. M. Dust , E. Buncel , J. Chem. Soc., Perkin Trans 2002, 2, 1627.

[34] D. Vichard , T. Boubaker , F. Terrier , J. M. Dust , E. Buncel , Can. J. Chem. 2001, 79, 1617.

[35] T. Boubaker , R. Goumont , E. Jan , F. Terrier , Org. Biomol. Chem. 2003, 1, 2764. 12948203 10.1039/b306437a

[36] F. G. Terrier , F. L. Debleds , J. V. Verchere , A. P. Chatrousse , J. Am. Chem. Soc. 1985, 107, 307.

