# Supplementary material for: Kinetic and Thermodynamic Study of Proton Transfer Reactions of 1‐Hydroxy‐2,2‐Dinitroethane: Establishing a Predictive Relationship between Tautomeric and Acidity Constants in Water
Source: Chemphyschem. 2025 Jul 23;26(17):e202500219. doi: 10.1002/cphc.202500219 (PMC12447123; doi:10.1002/cphc.202500219)
Supplement: Supplementary file 1 — Supplementary Material [file CPHC-26-e202500219-s001.pdf]

Kinetic and Thermodynamic Study of Proton Transfer Reactions of 1-hydroxy-2,2-dinitroethane: Establishing a Predictive Relationship Between Tautomeric and Acidity Constants in Water

Rania Khaldi, Amel Hedhli, Taoufik Boubaker*

Laboratoire de Chimie Hétérocyclique, Produits Naturels et Réactivité (LR11ES39), Faculté des Sciences de Monastir, Université de Monastir, Avenue de l'Environnement, 5019 Monastir-Tunisie.

***Authors to whom correspondence should be addressed**
Email: boubaker_taoufik@yahoo.fr

-Supporting Information-

Table of Contents

UV-Vis Absorption Spectra (**Figure S1**) 2

Effect of Buffer Concentration and pH on Deprotonation Kinetics:

Cacodylate Buffer (**Figure S2**) 2

Succinate Buffer (**Figure S3**) 3

Effect of Buffer Concentration and pH on Protonation Kinetics:

Formic Acid Buffer (**Figure S4**) 3

Methoxyacetic Acid Buffer (**Figure S5**) 4

Chloroacetic Acid Buffer (**Figure S6**) 4

Cyanoacetic Acid Buffer (**Figure S7**) 5

(pK_N) versus (pK_a^{CH}) Relationship (**Figure S8**)

Detailed Kinetic Data and k_{obsd} Values for all Investigated Reactions (**Tables S1–S7**) 6-9

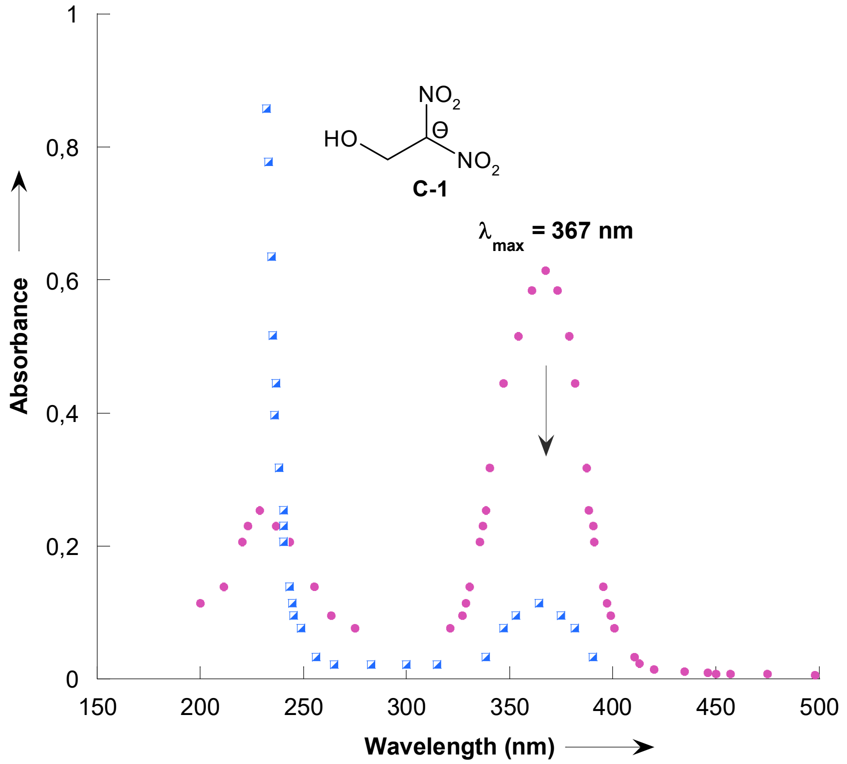

Figure S1. UV-Vis absorption spectra of 1-hydroxy-2,2-dinitroethanide **C-1** in aqueous solution at 25 °C and constant ionic strength of 0.1 mol L^{-1} maintained with KCl.

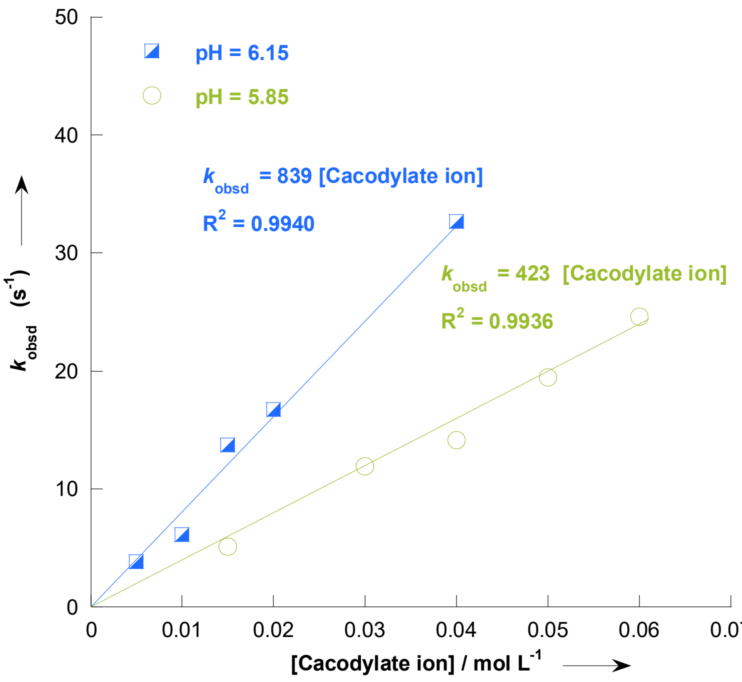

Figure S2. Effect of buffer concentration and pH on the observed rate constant, k_{obsd}, for the deprotonation of 1-hydroxy-2,2-dinitroethane **1** in cacodylate buffer in aqueous solution at 25 °C, with an ionic strength of 0.1 mol L^{-1} KCl.

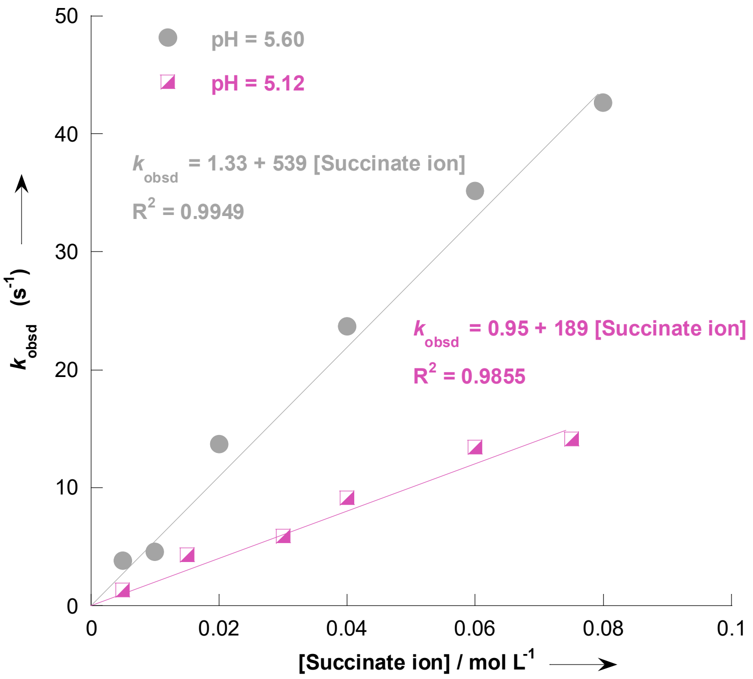

Figure S3. Effect of buffer concentration and pH on the observed rate constant, k_{obsd}, for the deprotonation of 1-hydroxy-2,2-dinitroethane **1** in succinate buffer in aqueous solution at 25 °C, with an ionic strength of 0.1 mol L^{-1} KCl.

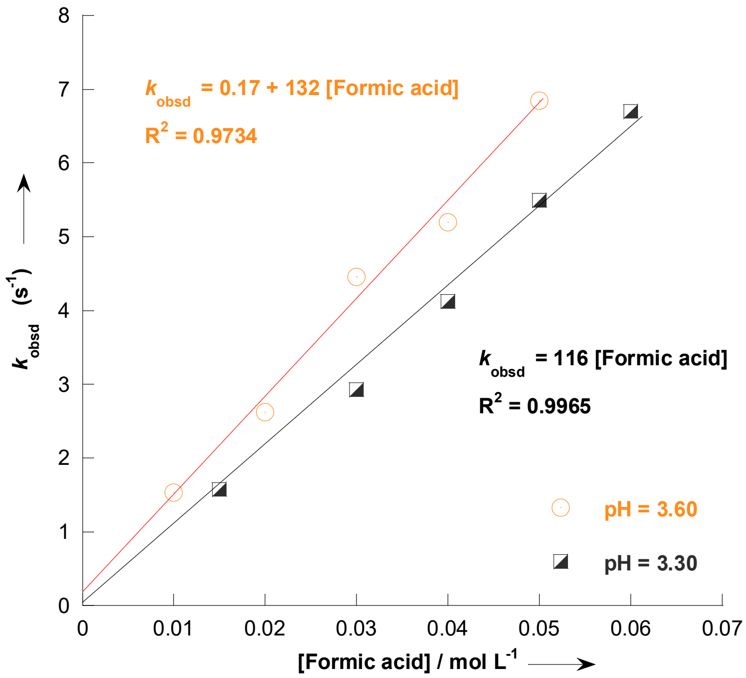

Figure S4. Effect of buffer concentration and pH on the observed rate constant, k_{obsd}, for the protonation of 1-hydroxy-2,2-dinitroethanide **C-1** in formic acid buffer in aqueous solution at 25 °C, with an ionic strength of 0.1 mol L^{-1} KCl.

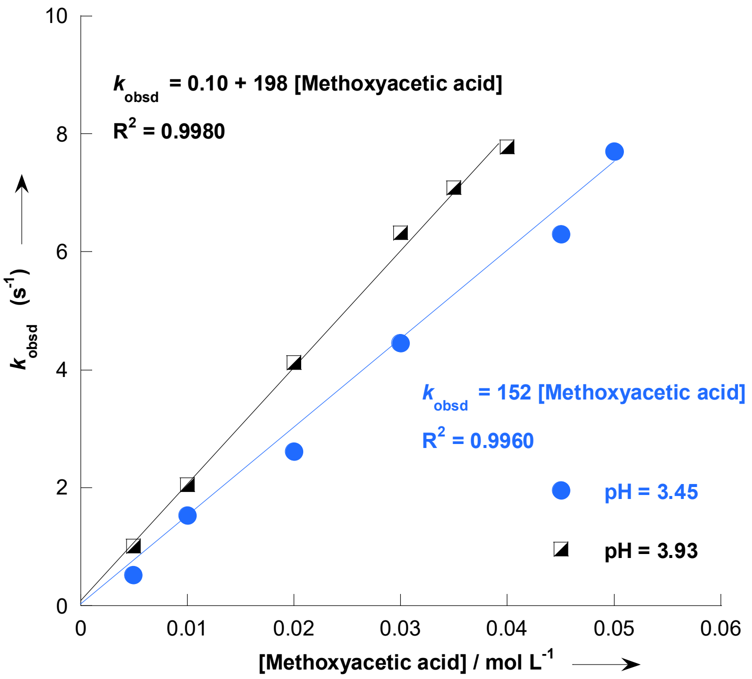

Figure S5. Effect of buffer concentration and pH on the observed rate constant, k_{obsd}, for the protonation of 1-hydroxy-2,2-dinitroethanide **C-1** in methoxyacetic acid buffer in aqueous solution at 25 °C, with an ionic strength of 0.1 mol L^{-1} KCl.

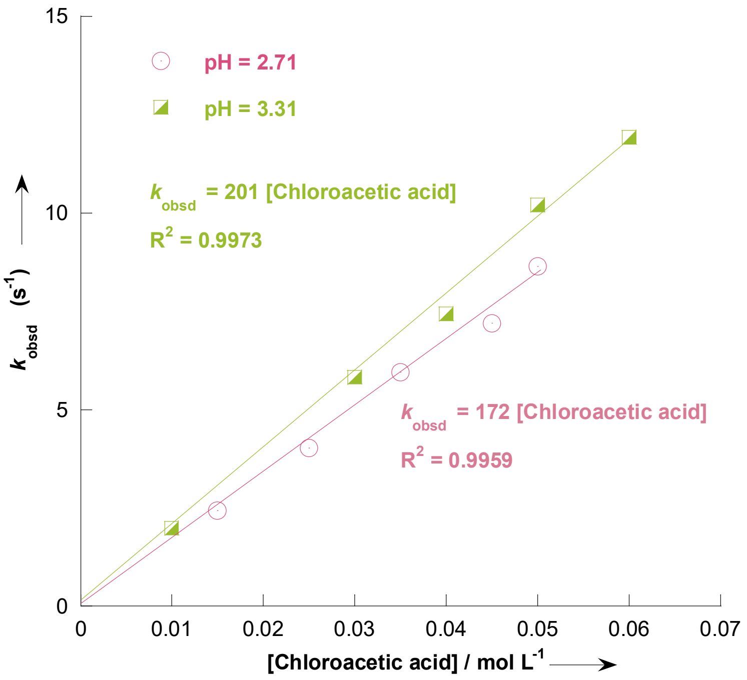

Figure S6. Effect of buffer concentration and pH on the observed rate constant, k_{obsd}, for the protonation of 1-hydroxy-2,2-dinitroethanide **C-1** in chloroacetic acid buffer in aqueous solution at 25 °C, with an ionic strength of 0.1 mol L^{-1} KCl.

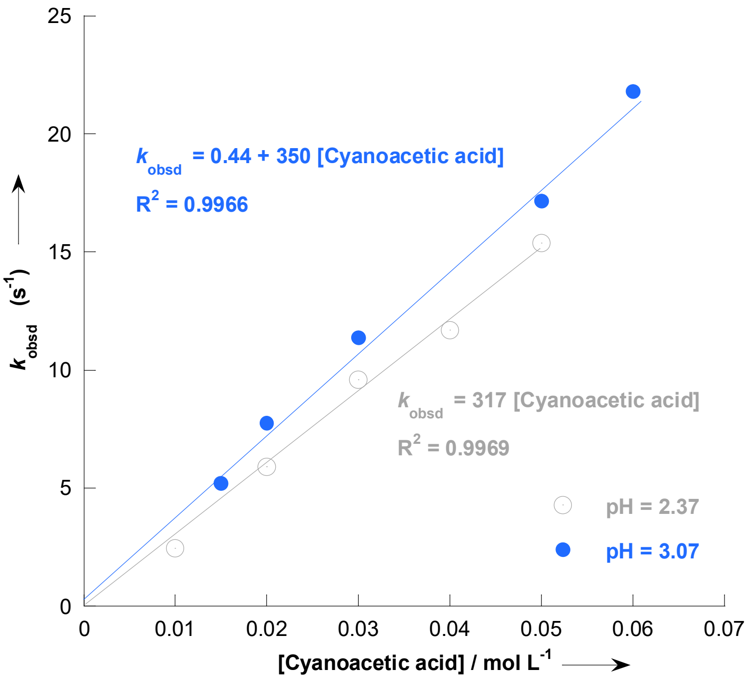

Figure S7. Effect of buffer concentration and pH on the observed rate constant, k_{obsd}, for the protonation of 1-hydroxy-2,2-dinitroethanide **C-1** in cyanoacetic acid buffer in aqueous solution at 25 °C, with an ionic strength of 0.1 mol L^{-1} KCl.

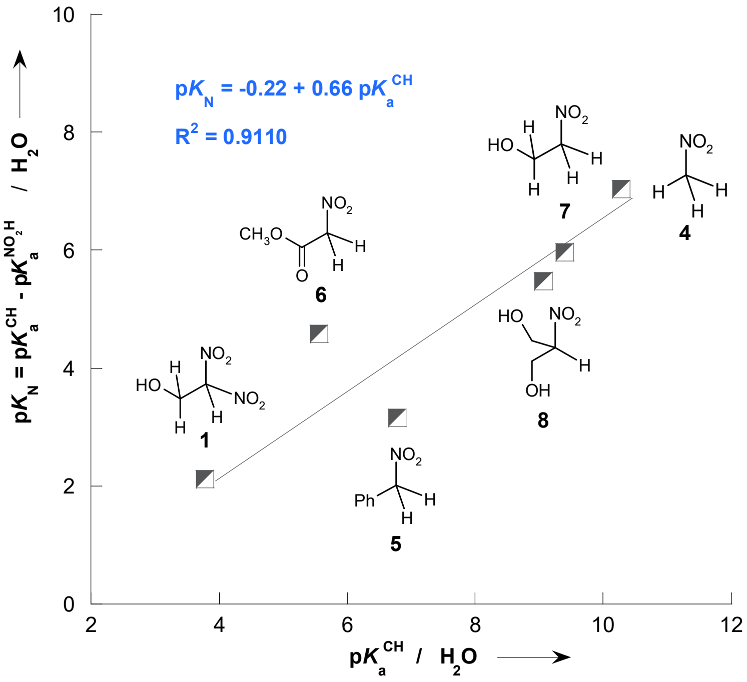

Figure S8. Plot of tautomeric equilibrium constant (pK_N) versus the acidity constant (pK_a^{CH}) for selected nitroalkanes **1** and **4–8** in water at 25 °C.

Table S1. Effect of buffer concentration and pH on the observed rate constant, k_{obsd}, for the deprotonation of 1-hydroxy-2,2-dinitroethane **1** in cacodylate buffer in aqueous solution at 25 °C, with an ionic strength of 0.1 mol L^{-1} KCl.

Cacodylic acid (pK_a = 6.15)			
[B]/[BH] = 1/1 (pH = 6.15)		[B]/[BH] = 1/2 (pH = 5.85)	
[B] (mol L^{-1})	k_{obsd} (s^{-1})	[B] (mol L^{-1})	k_{obsd} (s^{-1})
5×10^{-3}	3.83	1.5×10^{-2}	5.10
1×10^{-2}	6.11	3×10^{-2}	11.91
1.5×10^{-2}	13.72	4×10^{-2}	14.14
2×10^{-2}	16.73	5×10^{-2}	19.44
4×10^{-2}	32.68	6×10^{-2}	24.64

Table S2. Effect of buffer concentration and pH on the observed rate constant, k_{obsd}, for the deprotonation of 1-hydroxy-2,2-dinitroethane **1** in succinate buffer in aqueous solution at 25 °C, with an ionic strength of 0.1 mol L^{-1} KCl.

Succinate ion (pK_a = 5.60)			
[B]/[BH] = 1/1 (pH = 5.60)		[B]/[BH] = 1/3 (pH = 5.12)	
[B] (mol L^{-1})	k_{obsd} (s^{-1})	[B] (mol L^{-1})	k_{obsd} (s^{-1})
5×10^{-3}	3.82	5×10^{-3}	1.36
1×10^{-2}	4.61	1.5×10^{-2}	4.30
2×10^{-2}	13.72	3×10^{-2}	5.91
4×10^{-2}	23.73	4×10^{-2}	9.14
6×10^{-2}	35.20	6×10^{-2}	13.44
8×10^{-2}	42.70	7.5×10^{-2}	14.13

Table S3. Effect of buffer concentration and pH on the observed rate constant, k_{obsd}, for the protonation of 1-hydroxy-2,2-dinitroethanide **C-1** in acetic acid buffer in aqueous solution at 25 °C, with an ionic strength of 0.1 mol L^{-1} KCl.

Acetic acid (pK_a = 4.64)			
[B]/[BH] = 1/1 (pH = 4.64)		[B]/[BH] = 1/2 (pH = 4.34)	
[BH] (mol L^{-1})	k_{obsd} (s^{-1})	[BH] (mol L^{-1})	k_{obsd} (s^{-1})
5 x 10^{-3}	0.64	5 x 10^{-3}	0.55
1 x 10^{-2}	1.54	1 x 10^{-2}	1.00
2 x 10^{-2}	2.62	1.5 x 10^{-2}	1.38
3 x 10^{-2}	4.45	2 x 10^{-2}	2.13
4 x 10^{-2}	6.20	3 x 10^{-2}	3.13
5 x 10^{-2}	7.30	3.5 x 10^{-2}	3.49
-	-	4 x 10^{-2}	3.78

Table S4. Effect of buffer concentration and pH on the observed rate constant, k_{obsd}, for the protonation of 1-hydroxy-2,2-dinitroethanide **C-1** in formic acid buffer in aqueous solution at 25 °C, with an ionic strength of 0.1 mol L^{-1} KCl.

Formic acid (pK_a = 3.60)			
[B]/[BH] = 1/1 (pH = 3.60)		[B]/[BH] = 1/2 (pH = 3.30)	
[BH] (mol L^{-1})	k_{obsd} (s^{-1})	[BH] (mol L^{-1})	k_{obsd} (s^{-1})
1 x 10^{-2}	1.54	1.5 x 10^{-2}	1.58
2 x 10^{-2}	2.62	3 x 10^{-2}	2.93
3 x 10^{-2}	4.46	4 x 10^{-2}	4.12
4 x 10^{-2}	5.20	5 x 10^{-2}	5.49
5 x 10^{-2}	6.84	6 x 10^{-2}	6.70

Table S5. Effect of buffer concentration and pH on the observed rate constant, k_{obsd}, for the protonation of 1-hydroxy-2,2-dinitroethanide **C-1** in methoxyacetic acid buffer in aqueous solution at 25 °C, with an ionic strength of 0.1 mol L^{-1} KCl.

Methoxyacetic acid (pK_a = 3.45)			
[B]/[BH] = 1/1 (pH = 3.45)		[B]/[BH] = 3/1 (pH = 3.93)	
[BH] (mol L^{-1})	k_{obsd} (s^{-1})	[BH] (mol L^{-1})	k_{obsd} (s^{-1})
5×10^{-3}	0.53	5×10^{-3}	1.01
1×10^{-2}	1.54	1×10^{-2}	2.05
2×10^{-2}	2.62	1.5×10^{-2}	1.38
3×10^{-2}	4.45	2×10^{-2}	4.12
4.5×10^{-2}	6.30	3×10^{-2}	6.32
5×10^{-2}	7.70	3.5×10^{-2}	7.09
-	-	4×10^{-2}	7.78

Table S6. Effect of buffer concentration and pH on the observed rate constant, k_{obsd}, for the protonation of 1-hydroxy-2,2-dinitroethanide **C-1** in chloroacetic acid buffer in aqueous solution at 25 °C, with an ionic strength of 0.1 mol L^{-1} KCl.

Chloroacetic acid (pK_a = 2.71)			
[B]/[BH] = 1/1 (pH = 2.71)		[B]/[BH] = 4/1 (pH = 3.31)	
[BH] (mol L^{-1})	k_{obsd} (s^{-1})	[BH] (mol L^{-1})	k_{obsd} (s^{-1})
1.5×10^{-3}	2.43	1×10^{-2}	1.98
2.5×10^{-2}	4.02	3×10^{-2}	5.83
3.5×10^{-2}	5.96	4×10^{-2}	7.43
4.5×10^{-2}	7.20	5×10^{-2}	10.20
5×10^{-2}	8.64	6×10^{-2}	11.92

Table S7. Effect of buffer concentration and pH on the observed rate constant, k_{obsd}, for the protonation of 1-hydroxy-2,2-dinitroethanide **C-1** in cyanoacetic acid buffer in aqueous solution at 25 °C, with an ionic strength of 0.1 mol L^{-1} KCl.

Cyanoacetic acid (pK_a = 2.37)			
[B]/[BH] = 1/1 (pH = 2.37)		[B]/[BH] = 5/1 (pH = 3.07)	
[BH] (mol L^{-1})	k_{obsd} (s^{-1})	[BH] (mol L^{-1})	k_{obsd} (s^{-1})
1 x 10^{-2}	2.45	1.5 x 10^{-2}	5.23
2 x 10^{-2}	5.89	2 x 10^{-2}	7.78
3 x 10^{-2}	9.60	3 x 10^{-2}	11.38
4 x 10^{-2}	11.70	5 x 10^{-2}	17.16
5 x 10^{-2}	15.39	6 x 10^{-2}	21.81
